# Pediatric Intracranial Hypertension

**DOI:** 10.15844/pedneurbriefs-30-10-2

**Published:** 2016-10

**Authors:** Lalitha Sivaswamy

**Affiliations:** 1 Departments of Pediatrics and Neurology, Children’s Hospital of Michigan, Wayne State University School of Medicine, Detroit, MI

**Keywords:** Idiopathic Intracranial Hypertension, Disc Edema, Pseudotumor Cerebri

## Abstract

Investigators from the Ohio State University, Oregon Health and Science University and Rosalind Franklin School of Medicine examined the presenting manifestations, demographics and treatment strategies in children enrolled in the Intracranial Hypertension Registry (IHR).

Investigators from the Ohio State University, Oregon Health and Science University and Rosalind Franklin School of Medicine examined the presenting manifestations, demographics and treatment strategies in children enrolled in the Intracranial Hypertension Registry (IHR). A total of 203 children met the criteria for inclusion and data was analyzed for both primary (idiopathic) and secondary intracranial hypertension (PIH and SIH respectively).

The most common presenting symptoms were headache (over 95% in both groups) and visual symptoms (over 70% in both groups). Tinnitus was noted by slightly less 50% of subjects, while pain in neck/shoulders was present in over half of all children. Bilateral optic nerve edema was the physical finding noted with greatest frequency in all children (87%). Unilateral abducens palsy was present in 12% of children with PIH and 15% of children with SIH. Abnormalities on imaging were rare in PIH. However, those belonging to the SIH category were noted to have venous thrombosis in a significant minority. As expected, most children were treated with acetazolamide. Surgical procedures including placement of shunts and optic nerve sheath fenestration were surprisingly common in both cohorts (34% in those with PIH and 62% in those with SIH). Interestingly, about a third of children were diagnosed prior to puberty, with girls and boys being almost equally affected in this age group, whereas, in the post-pubertal cohort the disease predominantly affected girls. [1]

COMMENTARY. Despite descriptions of primary intracranial hypertension by Dandy as early as 1937, the pathogenesis of the disease remains obscure. Increase in venous sinus pressure and altered CSF dynamics have been postulated [2].

As pediatricians specializing in neurological disorders of childhood, one often hears the lament that children are not just diminutive adults and therefore registries and treatment trials that include children are of paramount importance. A case in point is in the case of primary intracranial hypertension (more widely referred to as pseudotumor cerebri or idiopathic intracranial hypertension, with the term “benign” intracranial hypertension being rightfully phased out). While most adults with PIH are indeed obese females, the IHR, clearly indicates that in young children the disease is equally common in boys. Further, the association with obesity in younger children remains tenuous. In pre-pubertal subjects, obesity was in fact, not a risk factor for PIH. This should draw the attention of clinicians to a sub-group of children who may not have been typical subjects for investigation of this condition.

The IHR establishes that the presence of bilateral optic disc edema remains the sine qua non of the neurological examination. Therefore, emphasis on physical examination findings is crucial despite the widespread use of imaging. In fact, fundus photography may be the ideal way to evaluate for disc edema, given the low proficiency of many medical providers in examining the fundus. Nonetheless, disc edema was not universal.

The measurement of opening pressure remains the cornerstone to establishing the diagnosis. Both the modified Dandy Criteria and the diagnostic criteria enunciated by Friedman et al. suggest that opening pressure above 28 cm of water be considered abnormal [3]. However, 48 children in the registry had opening pressures less than the suggested cut-off. This once again evokes the question of what constitutes normal opening pressure in children and how to best measure intracranial pressure in children.

Last but not least 86 of 203 children underwent surgical interventions. In the absence of evidence-based guidelines, it is not surprising that a variety of surgical options are being implemented. The long term outcome of such interventions compared to medical management alone is an area that calls for scrutiny.

In conclusion, the establishment, enrollment, and systematic study of children in the Intracranial Hypertension Registry represents a definitive step in the study of this rare yet unique condition.
